# A Hidden Shunt Behind Post-COVID-19 Dyspnea: Partial Anomalous Pulmonary Venous Connection

**DOI:** 10.7759/cureus.107458

**Published:** 2026-04-21

**Authors:** John Bajouka, Ziad R Affas, Johnathan Stephan, Patrick Alexander

**Affiliations:** 1 Internal Medicine, Henry Ford Health System, Southfield, USA; 2 Cardiology, Henry Ford Health System, Southfield, USA

**Keywords:** covid 19, oxygen saturation step-up, partial anomalous pulmonary venous connection, right heart catheterization, sinus venosus atrial septal defect

## Abstract

Persistent exertional dyspnea following SARS-CoV-2 infection is frequently attributed to post-viral pulmonary pathology. When symptoms are accompanied by right ventricular (RV) dilation and pulmonary hypertension without elevated left-sided filling pressures, alternative etiologies including congenital heart disease must be considered. A 52-year-old man with prior COVID-19 pneumonia presented with one year of progressive exertional dyspnea. Transthoracic echocardiography demonstrated preserved left ventricular systolic function with RV dilation. Right heart catheterization revealed mild precapillary pulmonary hypertension with a significant oxygen saturation step-up at the superior vena cava-right atrial level. Cardiac computed tomography angiography confirmed partial anomalous pulmonary venous connection (PAPVC) with a sinus venosus atrial septal defect. This case emphasizes the necessity of maintaining a broad differential in patients with persistent dyspnea following COVID-19, cautioning against anchoring bias toward post-viral sequelae. While initial suspicion focused on Group 3 pulmonary hypertension, the findings of RV dilation and a normal pulmonary capillary wedge pressure mandated further investigation. This suggests that the acute pulmonary insult may have physiologically unmasked a previously compensated, hemodynamically significant PAPVC. Ultimately, the integration of invasive hemodynamics, specifically identifying an oxygen saturation step-up, and multimodality imaging is essential to identify rare but correctable congenital anomalies in adults presenting with non-specific respiratory complaints.

## Introduction

The global SARS-CoV-2 pandemic has left a significant cohort of patients with lingering respiratory symptoms, often categorized as post-acute sequelae of COVID-19 [1]. While persistent exertional dyspnea is frequently attributed to post-viral parenchymal lung disease or deconditioning, the presence of right ventricular (RV) remodeling and pulmonary hypertension (PH) necessitates a more rigorous investigation. In many cases, acute respiratory stress can serve as a tipping point, unmasking previously compensated cardiac anomalies. Partial anomalous pulmonary venous connection (PAPVC) is a rare congenital malformation where one or more pulmonary veins drain into the systemic venous circulation or the right atrium rather than the left atrium. In adults, this creates a chronic left-to-right shunt that may remain asymptomatic for decades [2]. However, when combined with an acute pulmonary insult, the resulting shift can lead to symptomatic right heart failure. PH can vary between cardiac and pulmonary conditions, broadly classified into five groups based on underlying pathophysiology. In this context, distinguishing between intrinsic pulmonary vascular disease and flow-mediated causes, such as congenital left-to-right shunts, is critical, as management strategies differ significantly and may include curative surgical intervention. We present a case of a 52-year-old man whose persistent post-COVID-19 dyspnea was ultimately found to be caused by a PAPVC and an associated sinus venosus atrial septal defect (SVASD), highlighting the danger of anchoring bias in the post-pandemic diagnostic landscape.

## Case presentation

**Figure 1:**
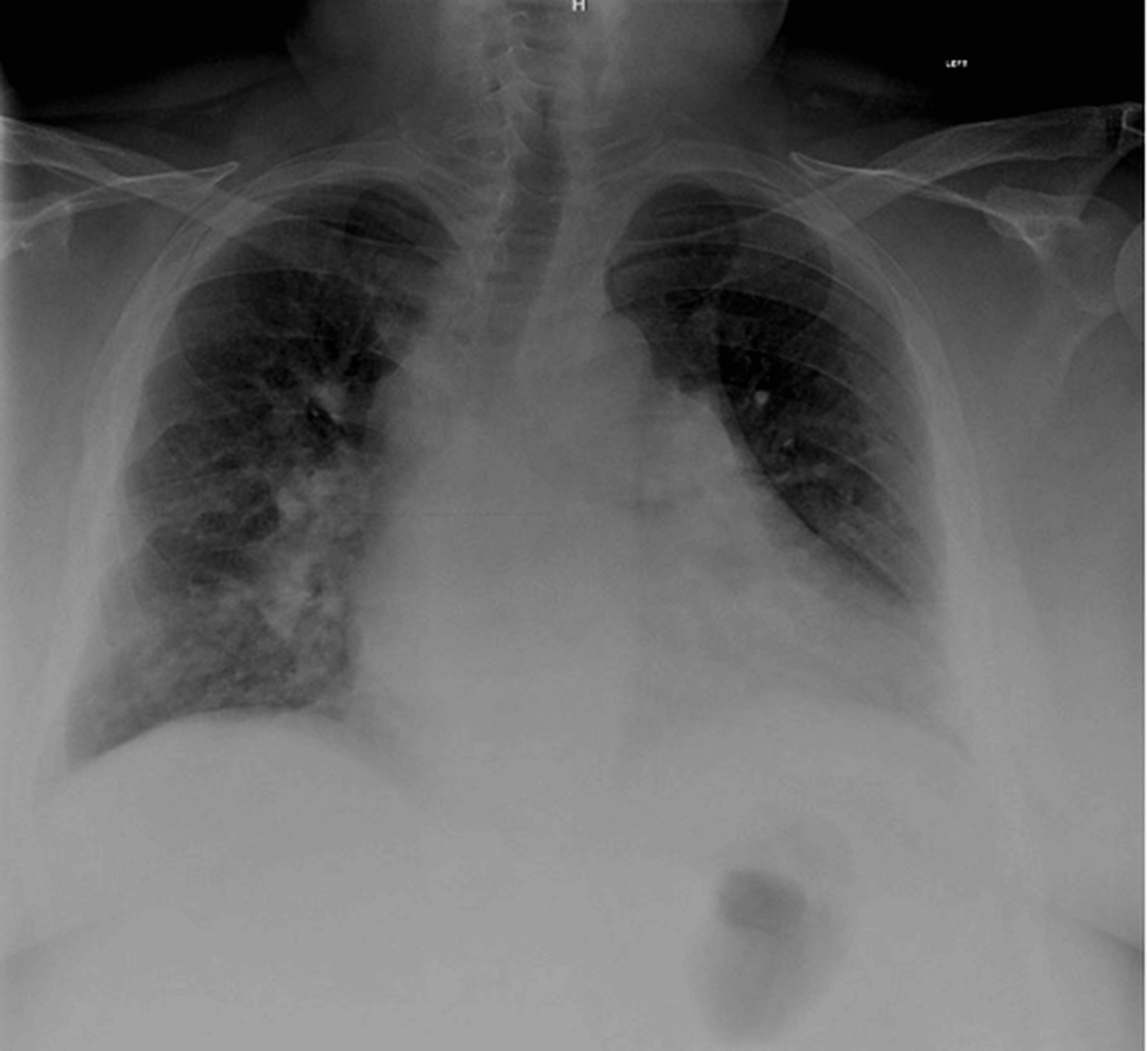
Chest X-ray Chest X-ray demonstrated stable cardiomegaly with no acute process.

A 52-year-old man presented with approximately 12 months of progressive exertional dyspnea accompanied by chronic non-productive cough and orthopnea. He denied chest pain, syncope, palpitations, or paroxysmal nocturnal dyspnea. The patient's past medical history included essential hypertension and prior COVID-19 viral pneumonia. Differential diagnosis included post-COVID-19 pulmonary disease, arrhythmogenic RV cardiomyopathy, chronic thromboembolic PH, idiopathic pulmonary arterial hypertension, and congenital heart disease with left-to-right shunt. On physical examination, the patient demonstrated signs of right-sided volume overload, including bilateral 2+ pitting lower extremity edema. Laboratory evaluation revealed normal cardiac biomarkers, including N-terminal pro-B-type natriuretic peptide (NT-proBNP) of 37 pg/mL (normal range: <300 pg/mL) and negative high-sensitivity troponins. An X-ray of the chest demonstrated stable cardiomegaly without acute pulmonary processes (Figure 1).

**Figure 2:**
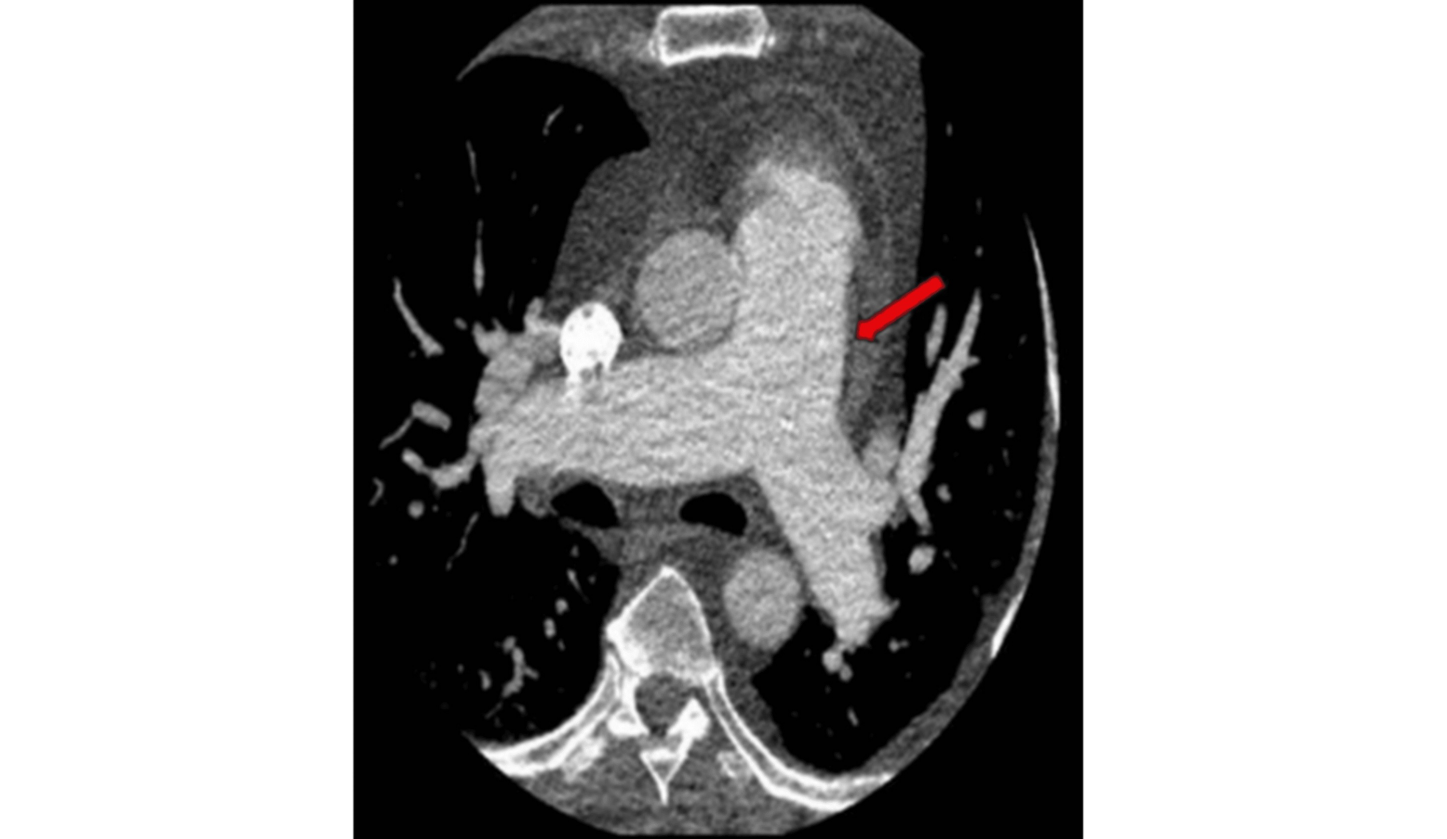
Chest computed tomography of the pulmonary arteries Chest computed tomography showing a dilated pulmonary outflow tract and main pulmonary arteries, compatible with increased pulmonary flow and volume.

**Figure 3:**
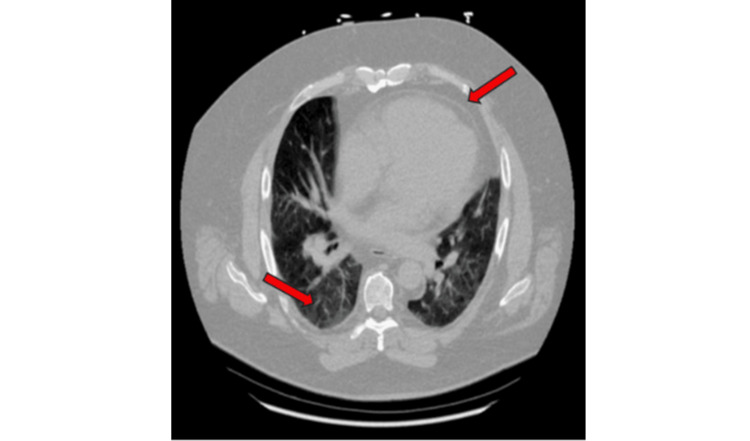
Chest computed tomography of the lung Chest computed tomography showing ground-glass opacities of the right lung and mild pericardial effusion.

Computed tomography (CT) was completed, which showed evidence of enlarged pulmonary arteries (Figure 2) and pulmonary venous congestion (Figure 3).

**Figure 4:**
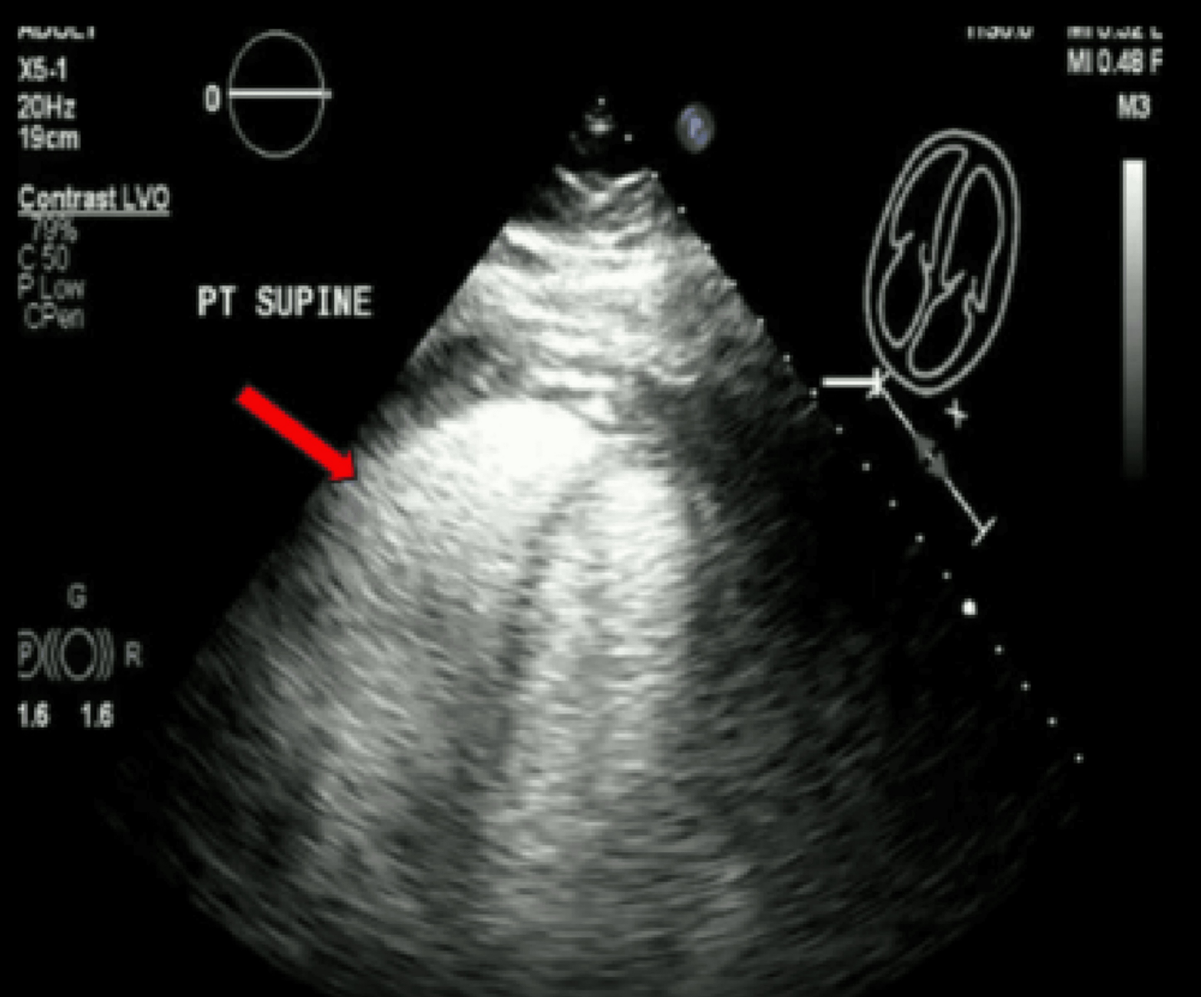
Transthoracic echocardiogram with contrast, 4-chamber view Transthoracic echocardiogram with contrast demonstrated a moderately dilated right ventricle with a preserved left ventricular size.

Transthoracic echocardiography (TEE) with contrast demonstrated a preserved left ventricular (LV) ejection fraction of 65%-70% with a moderately dilated, hypokinetic RV and systolic septal flattening (Figure 4).

Right heart catheterization (RHC) revealed right atrial pressure of 12 mmHg, mean pulmonary artery pressure of 31 mmHg, pulmonary capillary wedge pressure (PCWP) of 16 mmHg, cardiac output of 10.2 L/min, and pulmonary vascular resistance of 1.5 Wood units. Oxygen saturation measurements demonstrated a step-up from the superior vena cava (60.9%) to the right atrium (80.0%), a pulmonary-systemic shunt ratio of 2, consistent with a left-to-right shunt (Table 1). Pulmonary-to-systemic flow ratio (Qp/Qs) was 1.97, suggesting a significant left-to-right shunt.

**Table 1:** Right heart catheterization hemodynamics Summary of the findings of the right heart catheterization. Notably, an oxygen saturation step-up is observed between the SVC (60.9%) and the RA (80.0%), which is physiologically significant for a left-to-right shunt. RA: right atrium; RV: right ventricle; MPA: main pulmonary artery; PCWP: pulmonary capillary wedge pressure; SVC: superior vena cava; TPG: transpulmonary gradient; PVR: pulmonary vascular resistance; Fick: Fick principle for cardiac output calculation

Parameter	Value	Units
RA mean pressure	12	mmHg
RV	40/10	mmHg
MPA pressure	40/22	mmHg
Mean pulmonary artery pressure	31	mmHg
PCWP	16	mmHg
TPG	15	mmHg
PVR	1.5	Wood units
SVC oxygen saturation	60.9	%
RA oxygen saturation	80	%
MPA oxygen saturation	75.9	%
Aortic oxygen saturation	91.4	%
Cardiac output (Fick method)	10.2	L/min
Cardiac index	4.16	L/min/m²

**Figure 5:**
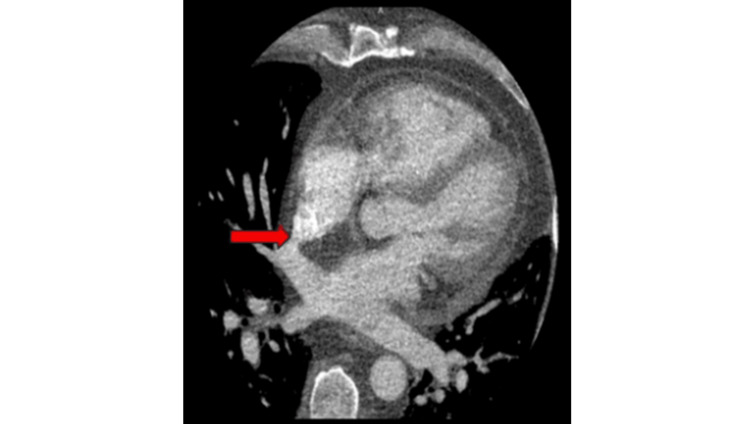
Cardiac computed tomography angiography Cardiac computed tomography demonstrated a partial anomalous pulmonary venous connection to the right atrium.

Given the physical examination findings of lower extremity edema and evidence of right heart failure, the patient was initially started on intravenous furosemide. He demonstrated a favorable clinical response with improvement in volume status and was eventually transitioned to an oral diuretic regimen. The patient’s persistent dyspnea and RV dilation prompted invasive hemodynamic evaluation. RHC subsequently revealed a significant left-to-right shunt evidenced by an oxygen saturation step-up. The elevated cardiac output in the setting of low pulmonary vascular resistance (1.5 Wood units) strongly suggested shunt physiology rather than intrinsic pulmonary vascular remodeling. Although the PCWP was mildly elevated at 16 mmHg, it was disproportionate to the degree of PH and did not account for the significant oxygen saturation step-up, effectively excluding isolated post-capillary PH. This suggested the PH was primarily related to the left-to-right shunt rather than Group 2 pathophysiology. Cardiac CT angiography (CTA) proved crucial in confirming PAPVC with an associated SVASD, a rare congenital anomaly often missed on standard echocardiography (Figure 5). This diagnosis shifted management toward surgical referral rather than solely medical therapy. Multimodality imaging combined with RHC findings was essential in guiding this accurate diagnosis and appropriate treatment plan.

Following a multidisciplinary heart team discussion, a surgical referral was placed for definitive repair. The planned operative approach is a Warden procedure to redirect the anomalous right pulmonary vein to the left atrium while preserving superior vena cava continuity and patency. The patient is currently awaiting surgical intervention.

## Discussion

This case illustrates a critical diagnostic pivot from presumed post-viral sequelae to a hemodynamically significant congenital defect, highlighting the importance of maintaining a broad differential diagnosis. A major learning point is the avoidance of anchoring bias, where clinicians may prematurely attribute persistent dyspnea and RV dilation solely to recent pulmonary insults, such as COVID-19. While post-viral PH is a valid concern, distinct hemodynamic markers, specifically in the setting of preserved LV function and borderline PCWP, should prompt a search for alternative etiologies beyond Group 2 or 3 PH.

COVID-19’s severe inflammation may have served as a physiological stressor that unmasked an otherwise compensated congenital shunt. Shifting pulmonary vascular resistance can worsen left-to-right shunting and precipitate symptomatic PH. This phenomenon underscores that adult-onset symptoms can represent the decompensation of a long-standing anatomical anomaly rather than a new pathology [3].

PAPVC remains a challenging diagnosis with a prevalence of 0.1%-0.2% in adults [4]. Because symptoms like dyspnea and arrhythmias are non-specific, it is frequently misdiagnosed as primary PH [5]. As demonstrated here, TEE provides initial clues, such as RV dilation, but often lacks the sensitivity to visualize the anomaly directly.

In this case, RHC was the critical bridge between anatomical suspicion and clinical intervention. While CT confirmed the presence of the anomalous vein, RHC quantified the physiological impact. The discovery of a significant oxygen saturation step-up in the superior vena cava and right atrium confirmed a significant left-to-right shunt. Importantly, the RHC demonstrated that the patient's PH was flow-mediated rather than resistance-mediated, as evidenced by a low pulmonary vascular resistance.

When clinical suspicion remains high due to unexplained RV overload, advanced imaging becomes mandatory. Cardiac CTA offers high spatial resolution independent of operator skill, making it superior to TEE for mapping anomalous veins [6,7].

Ultimately, this case serves as a reminder that rare, correctable causes of PH must remain in the differential for adults with non-specific pulmonary complaints. Integrating multimodality imaging with invasive hemodynamic assessment, specifically oxygen saturation step-up, is essential to look past common post-viral narratives and identify masked congenital shunts.

While many adults with PAPVC remain asymptomatic and may be managed conservatively with serial surveillance, surgical intervention is indicated when there is evidence of significant volume overload or functional impairment [8]. The decision-making process is partially reliant on the pulmonary-to-systemic flow ratio (Qp/Qs), which quantifies the shunt fraction using the Fick principle during RHC or via phase-contrast magnetic resonance imaging [9]. A pulmonary-to-systemic flow ratio of greater than 1.5 is widely considered the threshold for a hemodynamically significant shunt, as it correlates with progressive RV enlargement and eventual dysfunction [10]. Surgical correction utilizing techniques such as the Warden procedure, direct reimplantation of the anomalous vein into the left atrium, or intra-atrial baffling is recommended for symptomatic patients, those with pulmonary-to-systemic flow ratios of greater than 1.5, or those showing moderate or greater RV dilation even if asymptomatic [11]. Conversely, for patients with a pulmonary-to-systemic flow ratio less than 1.5 and no evidence of right heart strain, conservative management is appropriate, as these small shunts typically do not cause significant temporal deterioration in clinical or hemodynamic indices [8]. While the Warden procedure remains the surgical gold standard for redirecting anomalous flow, emerging data suggest that certain anatomical variants of PAPVC may now be amenable to percutaneous correction. Recent advancements in transcatheter techniques, including the use of covered endovascular stents to create an internal baffle, offer a minimally invasive alternative for select patients, potentially reducing the morbidity associated with open-heart surgery [12].

## Conclusions

In the context of post-COVID-19 cardiopulmonary symptoms, clinicians must maintain diagnostic vigilance for coexisting congenital anomalies, particularly when RV dysfunction and PH are present without elevated LV filling pressures. This case illustrates the power of multimodal diagnostics, where RHC-guided suspicion and cardiac CT confirmation led to the identification of surgically addressable PAPVC, transforming the therapeutic trajectory of the patient.
